# Structural Behavior and Fatigue of FRP-Reinforced Concrete Beams Exposed to Different Weathering Conditions

**DOI:** 10.3390/ma19050909

**Published:** 2026-02-27

**Authors:** Arash Rahmatian, Hussam Saleem, Farzad Hejazi, Michelle Nokken, Ashutosh Bagchi

**Affiliations:** 1 Department of Computer Science & Engineering, University of Houston-Downtown, Houston, TX 77002, USA; rahmatiana@uhd.edu; 2 Michael Baker International, 101 South Spring Street, Suite 100, Little Rock, AR 72201, USA; hsaleem@ualr.edu; 3 Faculty of Environment and Technology, The University of The West England, Bristol BS16 1QY, UK; 4 Department of Building, Civil & Environmental Engineering, Concordia University, Montreal, QC H3G 1M8, Canada; m.nokken@concordia.ca (M.N.); ashutosh.bagchi@concordia.ca (A.B.)

**Keywords:** GFRP, deflection, serviceability, CMOD, service limit state, fiber-reinforced polymer beam, crack mouth, cyclic loading over concrete beam

## Abstract

Fiber-reinforced polymer (FRP)-reinforced concrete beams are increasingly used in infrastructure, yet their flexural behavior under fatigue and harsh environmental conditions remains insufficiently studied. This study investigates the fatigue response and structural behavior of 12 glass-FRP (GFRP)-reinforced concrete beams under four environmental regimes: indoor control, continuous alkaline immersion, cyclic wet–dry alkaline immersion, and outdoor exposure in Montreal. Four pre-cracked beams were subjected to up to one million load cycles, while deflection and crack mouth opening displacement (CMOD) were monitored. Structural behavior was evaluated in terms of flexural capacity, load–deflection response, crack development (CMOD), stiffness degradation, and serviceability limit state (SLS) performance before and after fatigue loading. Results show that W&D and Immersion beams exhibited the largest deflections (δ_exp_/δc_ode_ = 158% and 92%, respectively), whereas Outdoor and Control beams maintained robust load capacity with minimal fatigue effect. Flexural toughness indices varied from 8.61 to 18.45 across specimens, highlighting environmental influence on energy absorption. Serviceability limit state criteria were reached between 400,000 and 850,000 cycles, depending on conditioning. Overall, GFRP-RC beams demonstrated strong residual strength and predictable degradation patterns, providing quantitative insight into fatigue performance under combined environmental and cyclic loading.

## 1. Introduction

Recent advancements in the field of fiber-reinforced polymer (FRP)-reinforced concrete (RC) beams have significantly enhanced our understanding of their fatigue behavior and durability under various environmental conditions. Jabbar et al. (2018) developed a correlation factor using particle swarm optimization to detect damage in Ultra-High Performance Fiber-Reinforced Concrete (UHPFRC) communication towers based on frequency data, demonstrating high accuracy and consistency between experimental and simulation results [1]. Building on early Carbon Fiber-Reinforced Polymer (CFRP) confinement studies, Ismail et al. (2019) found that partial CFRP confinement with horizontal strips at 20 mm spacing achieved comparable compressive strength to full confinement, noting that horizontal strips were more effective than helicoidal ones and highlighting limitations of existing confinement models, particularly for helicoidal configurations [2].

Pang et al. (2021) demonstrated that CFRP RC beams exhibit improved crack behavior and deflection compared to steel beams, although their load capacity is lower at high bar ratios, and noted that ACI 318-19 better predicts the moment redistribution in CFRP beams [3]. Shamass et al. (2022) studied Basalt Fiber-Reinforced Polymer (BFRP-reinforced concrete beams) and reported approximately 20% moment redistribution with improved durability, while noting that current design codes tend to overestimate capacities and underestimate deflections [4]. Zhao et al. (2022) found that high temperatures significantly weaken FRP-RC beams, increasing cracks and deflection and reducing bending capacity, with glass fiber FRP (GFRP) more affected than carbon fiber FRP (CFRP); their proposed models accurately predict residual performance [5]. Adimi et al. (1997) studied the fatigue behavior of GFRP bars embedded in concrete [6]. Janus et al. (2021) and Nagy et al. (2024) studied the fatigue behavior of GFRP-reinforced concrete beams [7,8]. Zhang et al. (2021) and Rahmatian et al. (2022) conducted extensive experimental studies demonstrating that FRP-RC beams exposed to freeze–thaw cycles, ultraviolet radiation, and alkaline environments exhibit notable degradation in mechanical performance, yet maintain superior fatigue resistance compared to traditional steel-reinforced beams [9,10].

Ortiz et al. (2023) [11] presented a review of FRP composites in concrete, noting enhanced material performance alongside limitations in flexibility and economic feasibility. They reported that environmental factors, particularly alkaline exposure, influence durability—with CFRP showing the greatest resistance—and identified distinctions in serviceability requirements between FRP-reinforced and steel-reinforced concrete. The study underscored the necessity for continued investigation into environmental effects and the development of design standards [11]. Shaikh and Sohoni (2023) investigated CFRP wrapping for damaged RC frames, showing that wraps, especially at 60° orientation and 1–2 mm thickness, effectively restore seismic performance, shear, and flexural capacity, while being lightweight, easy to install, corrosion-resistant, and more practical than traditional jacketing methods [12,13].

Luo et al. (2024) developed a simplified fatigue model for CFRP-strengthened RC beams under hot-wet and saline conditions, demonstrating that salinity significantly reduces fatigue limits more than temperature or humidity, providing insights for the design of coastal RC structures [14]. Cholostiakow et al. (2024) highlighted that beam depth reduces shear strength in FRP RC beams without shear reinforcement, whereas shear reinforcement mitigates size effects [15]. Guo et al. (2022) and Huang et al. (2024) further explored the effects of hygrothermal and saline environments, respectively, proposing simplified fatigue models that account for coupled environmental and cyclic loading conditions [14,16]. Tran et al. (2025) reviewed the bending response of FRP-reinforced concrete members, identifying gaps in AI/ML and discrete element analysis, limited research on deep and continuous beams, and recommending the use of ACI 440.1R for predicting moment capacity [17]. Zhang and Wang (2025) introduced soft computing techniques for predicting fatigue life, offering improved accuracy in modeling complex interactions between mechanical loading and environmental stressors [18]. Jia et al. (2025) applied advanced deep learning techniques, utilizing long short-term memory (LSTM) models, to estimate the fatigue lifespan of CFRP-reinforced beams equipped with fiber Bragg grating sensors, signifying progress toward smart structural health monitoring systems [19]. Jahani et al. (2024) investigated the fatigue behavior of near-surface-mounted CFRP-strengthened RC beams under elevated service temperatures, highlighting the importance of thermal effects on fatigue performance [20]. Recent reviews by Jahami and Issa (2023), Swaraj et al. (2025), and Osman and Elamin (2025) synthesized experimental and numerical findings, emphasizing the role of FRP layer configuration, anchorage systems, and failure modes in optimizing flexural and shear performance [21,22,23]. Collectively, these studies underscore the necessity of integrating environmental considerations into fatigue design frameworks and support the adoption of FRP as a sustainable alternative in reinforced concrete structures.

Although various FRP types such as CFRP, BFRP, and GFRP have been studied in the literature, the present study focuses specifically on GFRP-reinforced concrete beams. GFRP bars were selected due to their widespread use in civil infrastructure, cost-effectiveness compared to CFRP, and their higher susceptibility to environmental degradation, particularly under alkaline and moisture exposure, making them more representative for long-term durability and fatigue investigations. Previous studies on CFRP and BFRP-reinforced concrete beams are discussed to provide context and highlight general trends in FRP behavior; however, their material-specific differences are acknowledged, and direct comparisons are not the primary objective of this study. In this study, the term “structural behavior” refers to the global flexural response of FRP-reinforced concrete beams under combined environmental and cyclic loading, providing quantitative insights into fatigue performance and serviceability limit states that have not been reported in previous studies. A total of twelve specimens were constructed and after a 28-day curing period, four were intentionally pre-cracked to replicate service-level flexural damage and magnify subsequent environmental effects. The beams were subsequently exposed to one of the following condition regimes: interior laboratory control (Control), uninterrupted immersion in an alkaline environment (Imm), alkaline solution subjected to cyclic wet–dry conditions (W&D), and outdoor exposure under Montreal’s climate (Outdoor). The labels for specimens for fatigue testing were appended by “f” (e.g., Controlf, Immf, W&Df, and Outdoorf).

Previous studies have predominantly focused on externally bonded FRP sheets or plates, while limited experimental research has examined the fatigue behavior of concrete beams internally reinforced with GFRP bars under combined environmental exposure and cyclic loading. Moreover, most investigations emphasize ultimate strength rather than serviceability parameters such as deflection growth, CMOD, stiffness degradation, and fatigue damage stabilization.

This study presents a novel and comprehensive experimental investigation of full-scale GFRP-reinforced concrete beams subjected to coupled environmental conditioning and fatigue loading, with emphasis on serviceability-based performance assessment. A novel empirical relationship between deflection and fatigue cycles is established, and the stabilization stage of degradation is experimentally identified, providing new quantitative insight for durability-oriented design of GFRP-RC members.

Accordingly, this study aims to investigate the effect of environmental conditioning and cyclic loading on the structural response of GFRP-RC beams, focusing on key parameters such as load–deflection behavior, CMOD, flexural toughness, and SLS performance.

## 2. Specimen Design and Development of Reinforcement Configuration

**Figure 1:**
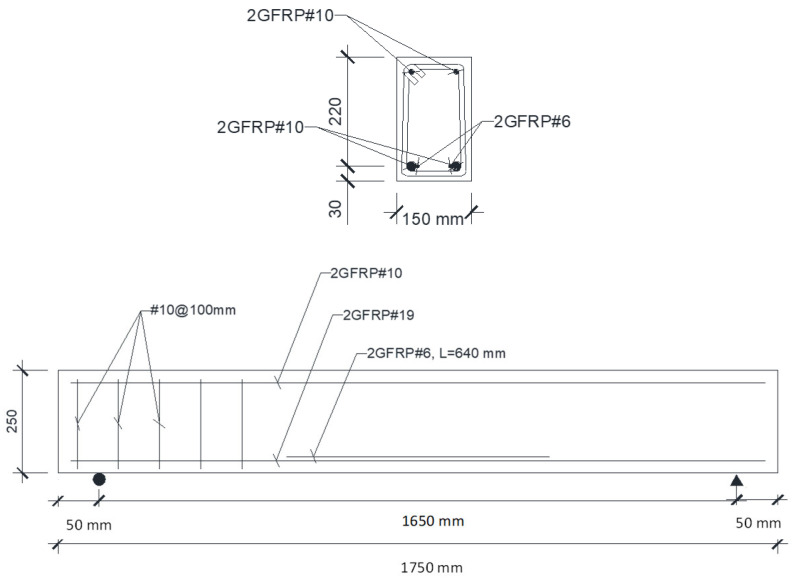
Profile and sectional representations of FRP-RC beams [26].

**Figure 2:**
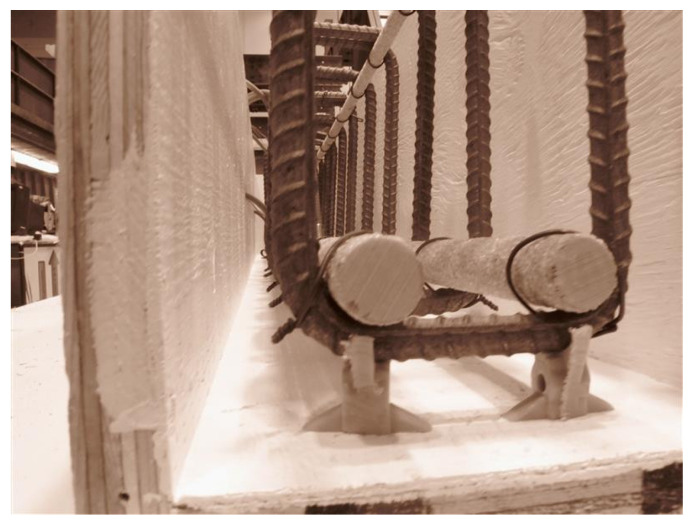
Profile specimen of FRP-RC beam.

The beams used as test specimens had a total length of 1.75 m and were developed in accordance with the guidelines provided in Intelligent Sensing for Innovative Structures Design Manual 31 (ISIS, 2007) [24]. The concrete was designed for a compressive strength of 35 MPa. GFRP bars were employed as both top and bottom rebars. The stirrups were formed using standard reinforcement of 10 mm diameter, arranged at one-hundred millimeters intervals throughout the beam span. GFRP bars were selected in this study due to their extensive use in civil infrastructure applications, particularly in bridge decks and beams, where corrosion resistance and cost efficiency are critical considerations. Compared to CFRP, GFRP offers a more economical solution, while exhibiting greater sensitivity to environmental exposure, especially alkaline and moisture-rich conditions. This characteristic makes GFRP a suitable material for investigating the combined effects of weathering and fatigue on long-term structural performance. GFRP reinforcement is characterized by linear elastic behavior up to failure, relatively high tensile strength, low modulus of elasticity compared to steel, and excellent resistance to corrosion. However, its mechanical properties can be influenced by environmental factors such as alkaline exposure, temperature variations, and moisture ingress, which may affect bond behavior, stiffness, and crack development in reinforced concrete members. The upper rebar consisted of two FRP bars of ten millimeters in diameter, while the lower reinforcement incorporated two bars of 19 mm diameter. The top reinforcement consisted of ten millimeter FRP bars, while the bottom reinforcement included 19 mm rebars. Supplemental FRP rebars with a diameter of six millimeters were instrumented with FOS strain sensors. According to Torkan (2010), the supplemental rebar should have a minimum length equal to twice its development length, and the diameter must remain less than that of the primary reinforcement so that the strain captured by the fiber-optic sensor corresponds closely to the strain experienced by the principal bar at the identical position [25]. The rebar dimensions adopted as part of this research conform to these recommendations. The beam and reinforcement layout are illustrated in Figure 1. Figure 2 shows FRP-RC beam specimens in the lab.

## 3. Experimental Program and Environmental Conditioning

The experimental program was designed to investigate the combined effects of environmental exposure and cyclic loading on the structural behavior of GFRP-reinforced concrete beams. Following casting and a standard curing period of 28 days under laboratory conditions, selected specimens were intentionally pre-cracked and subsequently subjected to different environmental conditioning regimes prior to fatigue testing.

### 3.1. Alkaline Conditioning

The alkaline environment was simulated using an aqueous sodium hydroxide (NaOH) solution with a pH of approximately 12.8, representative of the pore solution typically present in concrete. The solution temperature was maintained at laboratory ambient conditions (20 ± 2 °C). For the continuous immersion condition, specimens were fully submerged in the alkaline solution for the entire conditioning period. For the wet–dry (W&D) regime, specimens were subjected to repeated cycles consisting of alkaline immersion followed by air drying, simulating intermittent exposure commonly encountered in field conditions. The alkaline solution was periodically renewed to maintain a stable pH throughout the exposure duration.

### 3.2. Pre-Cracking Procedure

To replicate service-level flexural damage, four beams were intentionally pre-cracked prior to environmental conditioning. Pre-cracking was induced under monotonic four-point bending using a controlled loading protocol. Loading was applied until a target crack mouth opening displacement (CMOD) at mid-span was achieved, corresponding to the serviceability limit state. The load was then completely removed, ensuring that cracking remained within the elastic range of the GFRP reinforcement and that no concrete crushing or reinforcement failure occurred. This procedure enabled consistent initial crack conditions among pre-cracked specimens while avoiding irreversible structural damage.

### 3.3. Environmental Exposure Conditions

After pre-cracking, specimens were assigned to one of four exposure regimes. Control specimens were stored in an indoor laboratory environment at a temperature of 20 ± 2 °C and a relative humidity of approximately 50%. Immersion specimens were continuously exposed to the alkaline solution under controlled laboratory conditions. Wet–dry specimens underwent cyclic alkaline immersion and air-drying phases to simulate fluctuating environmental exposure. Outdoor specimens were placed in an open environment in Montreal, Canada, and subjected to natural weathering conditions, including seasonal temperature variations, precipitation, and freeze–thaw cycles. The duration and conditions of exposure were selected to represent realistic service environments for GFRP-reinforced concrete infrastructure.

## 4. Fatigue Loading Protocol and Cyclic Performance Evaluation of FRP-RC Beams

Bridge structures experience cyclic stresses from vehicular traffic, yet replicating their true response is challenging because of the complexity of load variations. Developing precise prototypes for comparison is both demanding and time-intensive, so researchers frequently adopt simplified load cycles to capture the essential fatigue behavior. Fatigue actions may vary in magnitude and frequency, but applying a constant amplitude and pattern provides a practical means of defining ultimate capacity under critical loading. This study reports experimental findings on FRP-reinforced concrete beams exposed to repeated cyclic loading of fixed load amplitude, evaluating the behavior of fiber-optic (FOS) and electrical strain gauge (ESG) sensors under different environmental exposures. The investigation also covers the beams’ serviceability limits and ultimate strengths.

The applied loading induced tension-tension stresses in the FRP bars, delivered as sinusoidal cycles at 2 Hz, with the minimum stress, $\sigma_{min}$, defined accordingly, Equation (1). This reflects the influence of additional loads applied to a bridge, such as pavement or installations, while the peak stress, *σ*ₘₐₓ, approaches roughly 80–90% of the ultimate strength capacity.

(1) $$\sigma_{mean}=\frac{\sigma_{min}+\sigma_{max}}{2}$$

**Figure 3:**
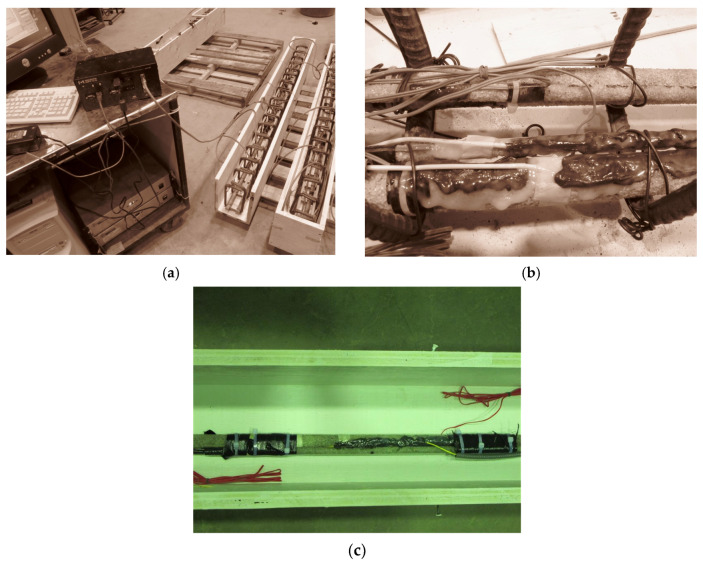
Experimental test setup for fatigue loading. (**a**) Preparation of specimens. (**b**) Installation wires and sensors on specimens [26]. (**c**) Instrumented beam before fatigue testing.

Actuator output and strain readings were recorded using the StrainSmart® 5000 Data Acquisition System manufactured by Vishay Precision Group, Wendell, NC, USA. collected at a frequency of 2 Hz. For each full loading–unloading cycle, parameters such as strain and deflection were documented to evaluate crack width and variations in deflection at intervals of 50,000 to 100,000 cycles, continuing up to one million cycles, with detailed discussion provided later. Figure 3 shows the experimental test setup for fatigue loading.

## 5. Bending Strength and Load-Bearing Ability

The energy required for fracture, also referred to as bending toughness, is a crucial factor influencing both the strength and ductility of a structure. In reinforced concrete FRP systems, it serves as a practical measure for comparing energy absorption and fracture resistance; however, uncertainties remain regarding its proper measurement, interpretation, and application [27]. The influencing factors comprise the loading rate, specimen size and geometry, loading configuration, machine stiffness, and the chosen method of interpretation. However, there is no standard method for the calculation of the flexural toughness in FRP-Reinforcement concrete beams, but by applying the concepts in ASTM and JSCE this index can be utilized for comparison cases. Bending rigidity can be defined using unitless metrics, as outlined in ASTM C1018 standard (ASTM International, 2003), that account for geometric effects, or by JSCE-SF4 (JSCE, 1984), which defines toughness in absolute terms independent of geometry [28,29]. According to ASTM C1018-03, the standard procedure for measuring flexural toughness involves dividing the area under the force–displacement curve up to a defined deflection based on the enclosed area corresponding to the displacement at which the first crack is considered to appear. Since determining deflection at the onset of cracking is both complex and debated, this procedure was removed from the code in 2006 owing to its limited precision and minimal use. In this investigation, flexural toughness was assessed through an adapted method, defined as the ratio between the energy dissipated at the serviceability threshold and that at the ultimate loading stage. This approach can serve to compare how various conditions impact structural reliability and resilience under extreme loads, creep, seismic events, and stress relaxation when deflection exceeds allowable limits. In this method, neither crack size nor crack count is considered. Instead, serviceability limits and ultimate bearing load are used as the primary criteria, making the definition applicable even to fatigued specimens that have already developed cracks.

*I* = *I*_ult_/*I*_all_ (2)

**Figure 4:**
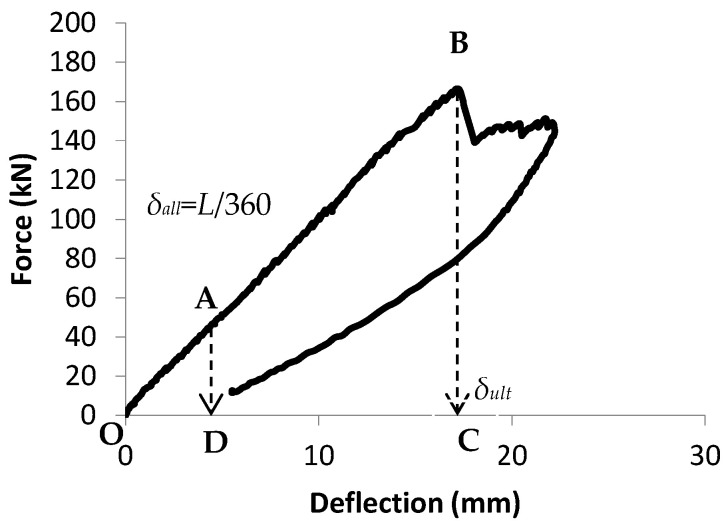
Calculation of flexural toughness [26].

Here, *I* refers to the bending toughness parameter; *I*_ult_ corresponds to the enclosed region beneath the load displacement graph till the ultimate force, denoted as *A*_ult_ (the region OBC in Figure 2); *I*_all_ represents the surface under the load displacement graph till the permissible displacement, corresponding to *A*_ult_ (region bounded by OBC in Figure 2); and *I*_all_ represents the area under the load–displacement graph till the allowable displacement, corresponding to *A*_all_ (region bounded by OAD in Figure 2) for serviceability (Figure 4). Table 1 shows the flexural toughness index.

Although Control1 exhibited the greatest ultimate load capacity, its toughness index was the lowest, recorded at *I* = 8.61. This observation also applies to the Controlf specimen, which exhibited the highest deflection at ultimate load among the fatigue-loaded beams, with toughness index reaching its lowest value of 13.17. Therefore, under the current assumptions, the control condition results in the lowest flexural toughness. The Imm1 and Imm2 specimens exhibited markedly different toughness index values, 9.12 and 18.45 respectively, which may be attributed to variations in their failure modes. The Immf specimen, exposed to fatigue loading, also exhibited a low toughness index of 13.93. The toughness parameter variation between W-D1 and W-D2 specimens is evident, whereas the test samples exposed outdoors exhibit comparable measurements for both static and fatigue conditions. As indicated in Table 1, weathering appears to enhance the toughness index and energy absorption capacity. However, under alkaline exposure, the extent of improvement shows greater variability.

## 6. Deflection

The peak deflection (δ_exp_) at failure load was recorded from the test and compared with the deflection limit recommended by ISIS (2007), denoted as δ_code_. The effective moment of inertia, *I*_e_, can be calculated from Equation (3). Table 2 lists the displacement ratios (δ_exp_/δ_code_) for all specimens. Among them, the specimen exposed to the wet–dry (W&D) condition shows the largest deviation from the allowable maximum deflection, δ_code,_ compared to the Control specimen.

(3) $$I_{e}={\left(\frac{M_{cr}}{M_{a}}\right)}^{3}\beta_{b}I_{g}+\left[1-{\left(\frac{M_{cr}}{M_{a}}\right)}^{3}\right]I_{cr}\leq \;I_{g}$$

**Figure 5:**
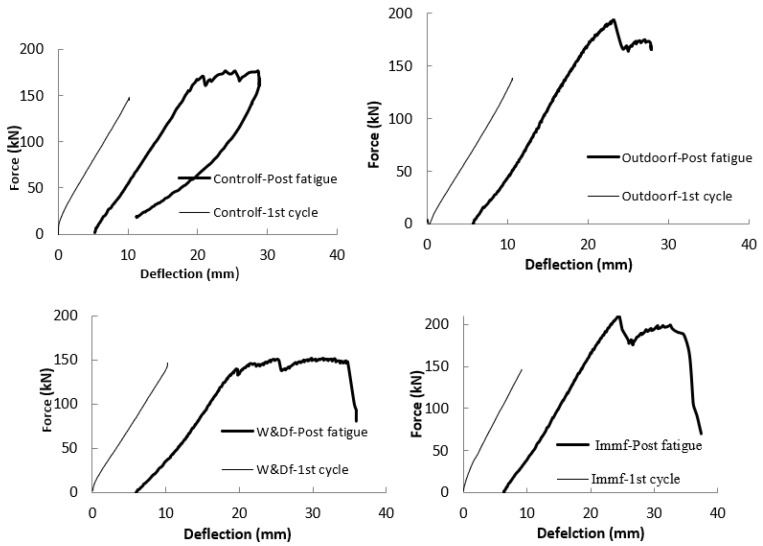
Effect of fatigue under four different conditions [26].

Figure 5 presents the force–displacement graphs obtained from post-fatigue static tests. In the plots, the thin lines represent the force–displacement response after one million fatigue cycles, while the thick lines correspond to the first-cycle of loading at a stress level of σ_mean_, as defined in Equation (1). In all cases, the rising portions of both curves run parallel to each other, with the thick line exhibiting a condition-specific offset that reflects the accumulated mid-span deflection. This pattern indicates that FRP-reinforced concrete beams maintain a strong load-bearing capacity under fatigue loading across all tested conditions, up to their ultimate strength.

## 7. Static Load Capacity After Fatigue Cycles

**Figure 6:**
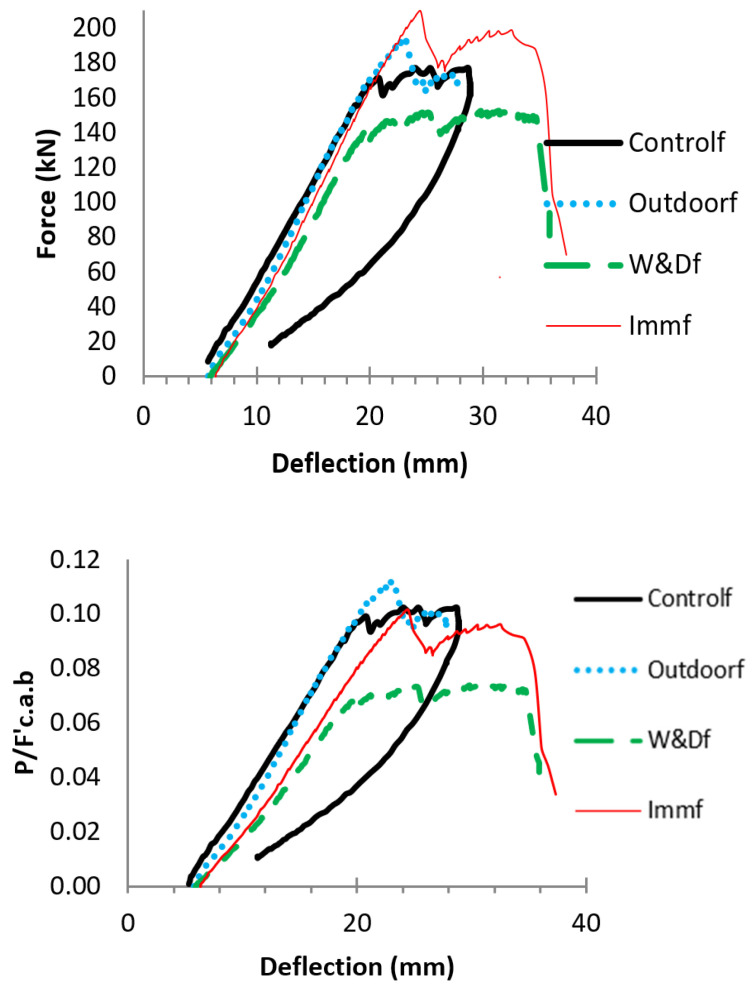
Force–deflection curves of beams under static testing after 1 m fatigue cycles: Top of the graphs, force versus displacement; Bottom—non-dimensional force versus displacement [26].

To evaluate the impact of weathering and fatigue on the beams’ load-carrying capacity, the post-fatigue specimens were subjected to static loads increasing monotonically until failure. The residual load-bearing capacities of the fatigued beams were subsequently compared to the capacities of equivalent samples that had not undergone prior fatigue loading. Figure 4 illustrates the force–displacement responses of the 4 specimens tested after 1,000,000 fatigue cycles. Overall, fatigue combined with exposure to alkaline solutions or environmental weathering can lead to bonding and internal failure, reducing the concrete aggregate’s interlocking strength. Among the specimens, Outdoorf exhibited the maximum flexural strength and greater deformability than Controlf, comparable to the behavior observed in W&Df and Immf (Figure 6).

**Figure 7:**
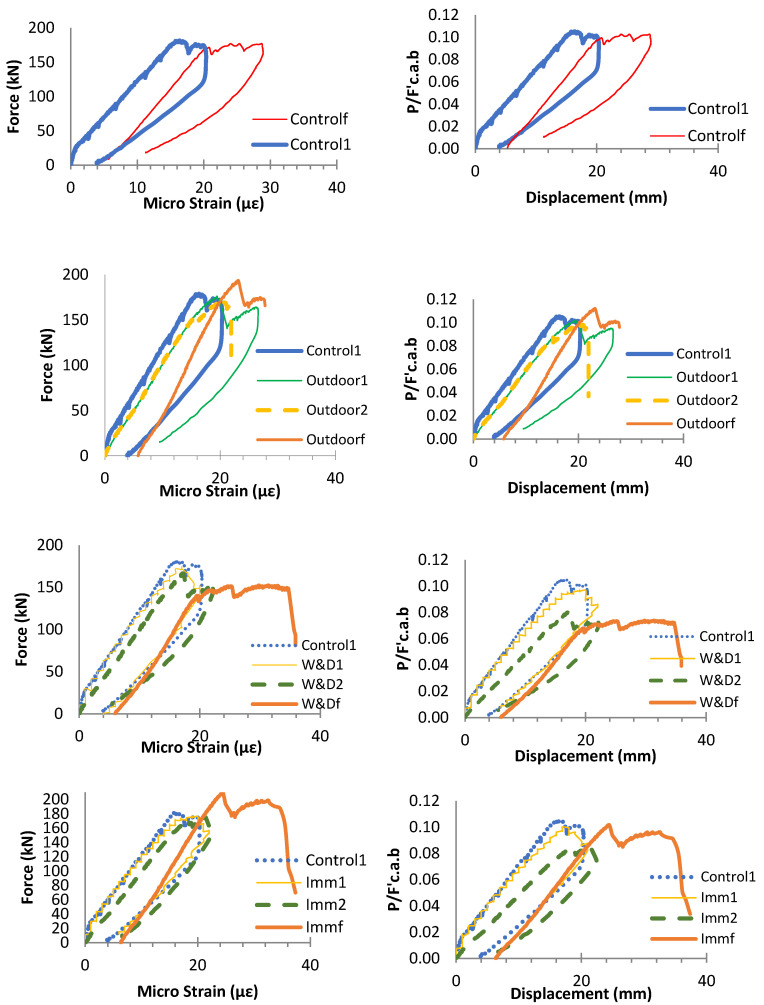
Beam load deflections for W&D and Immersion conditions: force–displacement (**Left**); non-dimensional load vs. deflection (**Right**) [26].

Figure 7 presents the load–deflection curves for each post-fatigue specimen, alongside Control1 and the corresponding samples that were not exposed to cyclic forces. Regarding flexural performance, the results indicate that cyclic loading had little influence on the Control and Immersion, and beams exposed to Outdoor conditions. In contrast, the W&Df sample showed noticeable fatigue-induced structural degradation. Experimental observations reveal that W&Df exhibited the lowest flexural resistance, failing in a balanced mode according to shear vs. compression under loading of static after fatigue, whereas the other samples predominantly failed in compression mode.

## 8. Influence of Fatigue Loads on Displacement

**Figure 8:**
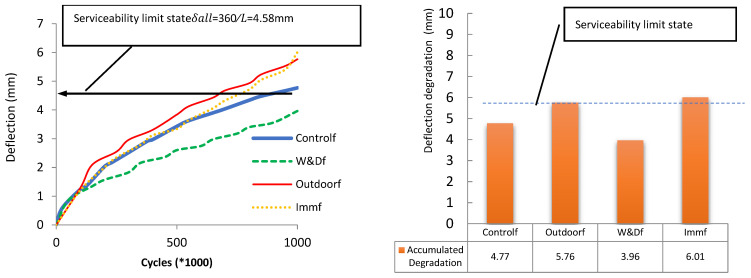
Deflection Behavior Across Fatigue Scenarios; Comparison of accumulated degradation in different conditions [26,29].

Displacement in beams and girders serves as a key serviceability criterion. Across all examined conditions, the displacement surpassed the (SLS) serviceability limit state of δₛ = L/360, as recommended by Intelligent Sensing for Innovative Structures Guidelines (ISIS 2007) [25], after approximately 600,000 cycles. For the fatigue-tested specimens, the calculated SLS deflection was 4.58 mm, as illustrated in Figure 6. The fatigue cycles corresponding to this threshold were determined as 650,000 for outdoor exposure, 750,000 under Imm conditioning, resulting in a value of 850,000 for the control specimen, and beyond 1,000,000 cycles for the W&Df condition. Among these, the W&Df specimen demonstrated the most favorable behavior within meeting the SLS requirement. Crack mouth opening displacement (CMOD) was monitored at intervals of 50,000 to 100,000 load cycles, while mid-span deflection was recorded concurrently. Figure 8 further shows the accumulated degradation obtained by summing the mid-span deflection measurements at intervals of 50,000 or 100,000 cycles throughout the entire loading sequence.

**Figure 9:**
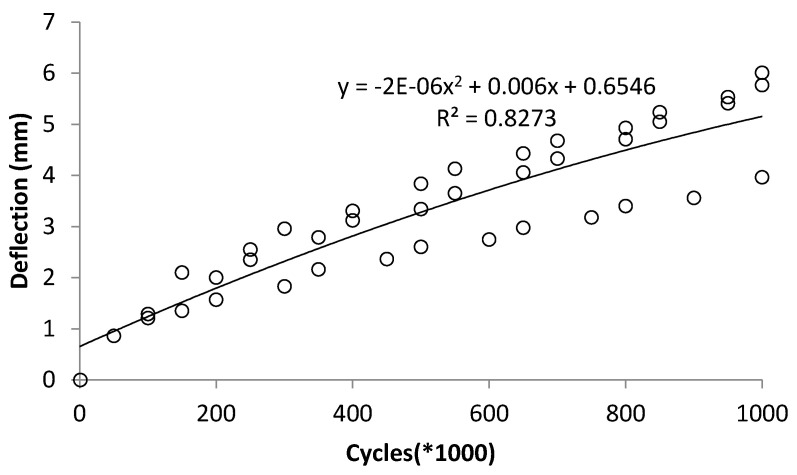
Fitted graph to relate the change in displacement because of fatigue cycles, accounting for all scenarios [30].

A practical technique for estimating the terminal degradation level under fatigue cycles is to identify the stabilization point on the deflection–cycle curve, where the slope approaches zero. At this stage, the beam has reached its maximum deterioration from cyclic loading and is unlikely to degrade further, though sudden failure may occur if loading continues. The middle of the span displacement of the beam was plotted compared with the fatigue cycles for all samples, along with the fitted graph, which produced a regression performance index, R^2^ equal to 0.82. Additional testing with extended cycles would be required to establish more data points near the stabilization or turning region of the graph, enabling a trend line with refined characteristics to better predict degradation rates. Based on Figure 9, the stabilization point is identified at approximately 1,500,000 cycles, where the gradient of the fitted graph (dy/dx) becomes 0. In such a case, the mid-span deflection and CMOD continue to increase with the number of fatigue cycles. This indicates progressive stiffness degradation due to crack propagation and bond weakening between GFRP bars and concrete. However, the rate of increase diminishes after a certain number of cycles, suggesting that the beam approaches a stabilization point in its fatigue response.

## 9. Displacement Response as an Indicator of Fatigue Damage

**Figure 10:**
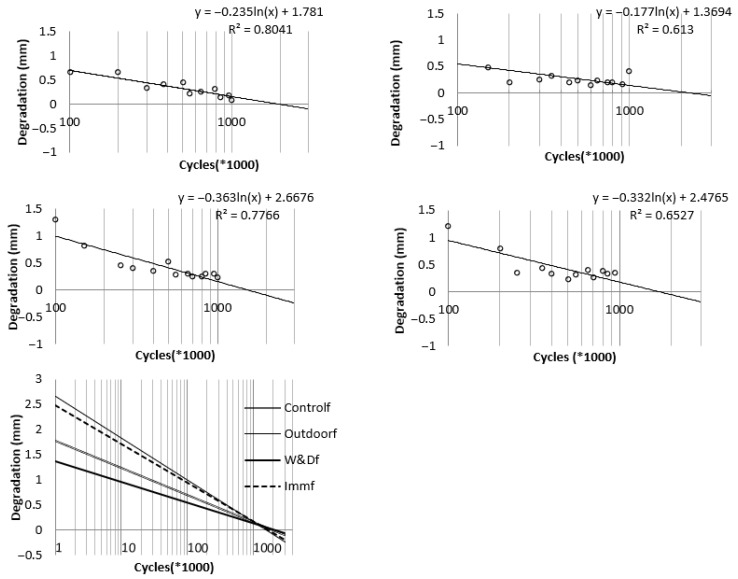
Decline in the Controlf, W&Df, Outdoorf, Immf conditions respectively from top to the bottom and left to right, last figure for trend curves showing the decline in different specimens [30].

For each condition examined, the degradation trend or best-fit line describing the variation in transverse displacement relative to the fatigue cycle count was determined and plotted on a semi-logarithmic scale, as illustrated in Figure 10. The compiled graph presents the degradation curves for all specimens. The extent of degradation was evaluated at 50,000-cycle intervals by determining the difference between the highest and lowest deflections measured by the actuator from the initial cycles to the final ones. Beam deterioration, expressed as increased deflection because of fatigue behavior, is denoted by *Dᵢ* (mm) for each case, with *N* representing the cycle count. The fitted graphs for the different conditions are represented by Equation (4), and the coefficients “a” and “b” obtained for each case are summarized in Table 3.

(4) $$D_{i}=a[\ln(N)]+b$$

Figure 10 shows that the deflection rate under fatigue loading gradually decreases as the number of loading cycles increases, eventually approaching zero. The cycle count at which this rate becomes zero—representing the turning point in the deflection–cycle curve—marks the stabilization stage, where the beam ceases to undergo further degradation from cyclic stresses. Prior to stabilization, reductions in strength and stiffness occur due to crack growth, diminished interlock and friction between aggregates, and weakening of the bond between concrete and reinforcement. Once these mechanisms can no longer progress, deflection stabilizes. The maximum cycle number for stabilization, *N*s, is defined by the intersection of a specimen’s degradation trend line with the horizontal axis. Based on Figure 10, *N*s was calculated as 2,100,000 cycles for W&Df, 1,700,000 million cycles for Immf, 1.5 million cycles for Outdoorf, and 1.8 million cycles for Controlf.

## 10. Prediction of Deflection and Cracking at the Serviceability Limit State (SLS)–Control

**Figure 11:**
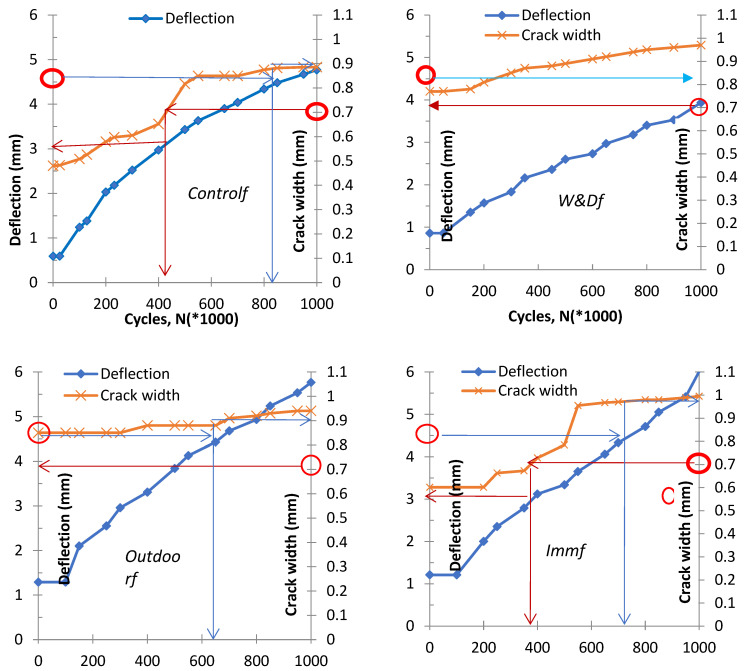
Forecast of serviceability limit states deflection and crack width in Controlf, W&Df, Outdoorf, Immf respectively, from top to bottom and left to right [26,29].

Equation (4) was applied to estimate deflection for each condition, with coefficient values listed in Table 3. Figure 11 illustrates the variation in displacement and the width of the crack with fatigue cycles, which can be used to determine the cycle counts in relation to serviceability limits. Experimental data showed that crack widths in all cases stabilized between 0.9 and one millimeter. The serviceability limit states (SLS) for displacement (e.g., L/360) and the width of the crack (e.g., 0.71 mm per ACI 440.1R-06) were adopted to identify the limiting fatigue cycles [31]. With the highest crack width limited to 0.7 mm as per ISIS (2007), the Controlf specimen sustained over 420,000 cycles with a deflection close to 3 mm [23]. Conversely, under control conditions, extrapolation indicated that the peak number of cycles corresponding to the deflection serviceability limit states of 4.58 mm was 800,000, at which point the crack width reached 0.9 mm.

**Figure 12:**
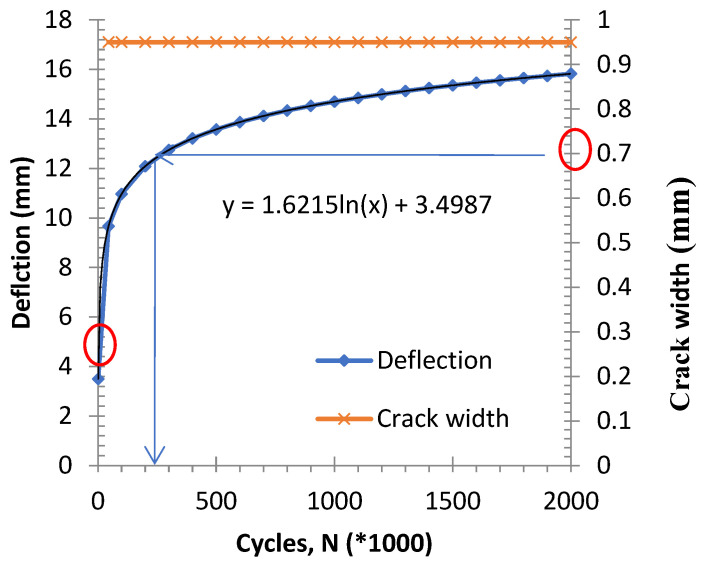
Estimation of the number of cycles by SLS as a combination of 3 criteria [26].

The progression of displacement and crack width under cyclic loading across diverse conditions shows how fatigue cycle restrictions are set based on SLS guidelines, and the results are summarized in Table 4. For the W/D fatigue-conditioned specimen, neither deflection nor crack width reached the SLS threshold, suggesting an extended fatigue life. In the Outdoorf specimen, crack serviceability was maintained, but at 650,000 cycles, the deflection SLS was met while the crack width surpassed the limit at ~0.9 mm. The Immf sample obtained the limiting width of the crack at 400,000 cycles with deflection below the limit, whereas considering the serviceability limit states deflection, the crack width increased to 0.95 mm at 750,000 cycles. Figure 12 presents these variations for W&D, Outdoor, and Immersion conditions, selected for their comparable mean stress values and closer simulation of real scenarios. Results further suggest that maximum crack width stabilizes near 0.9 mm, with the highest cycle count observed at 250,000 cycles, which is aligned with both serviceability limit states, deflection and crack width.

## 11. Summary

The objective of this study is to investigate the fatigue response and flexural behavior of GFRP-reinforced concrete beams under monotonic and cyclic loading, considering four environmental conditioning regimes: indoor laboratory control, continuous immersion in alkaline solution, cyclic wet–dry immersion, and outdoor exposure. The performance of these specimens is compared in terms of deflection, crack development (CMOD), stiffness degradation, and serviceability limit state (SLS), providing clear insight into the influence of environmental effects on GFRP-RC beams. The key outcomes for control and conditioned specimens exposed to one million fatigue cycles followed by monotonically increasing static loading till failure are summarized below.

- Experimental strains remained within the FRP serviceability limit (2000 με) at the SLS displacement criterion of L/360, which is critical under adverse conditions such as Outdoor, Immersion, or Wet& Dry exposure. Regarding flexural performance, cyclic loading had minimal impact on Control, Imm, and Outdoor specimens, with less than 5% reduction in stiffness. The Immf and W&Df beams exhibited greater deformability, showing approximately 15–20% higher mid-span deflection compared to Controlf and Outdoorf, but lower ultimate load by around 10–12%.
- Degradation was primarily due to environmental conditioning (alkaline exposure) rather than fatigue, with fatigue effects in Immersion and Outdoor specimens being negligible (<5%). Cracks from cyclic loading did not penetrate the compressive zone of the beams. Overall, FRP-RC beams demonstrated robust load-carrying capacity under fatigue throughout all conditions, reaching ultimate loads comparable to un-fatigued specimens (within 95–98% of peak strength).
- For serviceability deflection limits, the Immersion specimen performed worst, reaching SLS at ~750,000 cycles, whereas W&Df showed the best performance, with SLS not being reached even beyond 1,000,000 cycles. A fitted curve was developed to relate deflection changes to fatigue cycles across all criteria, and an empirical formula was established for degradation, considering all cases.
- Fatigue cycles corresponding to SLS criteria for crack width and deflection were determined from observed patterns. The crack and deflection SLSs occurred at approximately 800,000 and 1,100,000 cycles, respectively. Crack widths at 2000 με were 0.6 mm for Controlf, 0.4 mm for Outdoorf, and 0.2 mm for both Immf and W&Df. The reduced crack width in alkaline conditions is attributed to decreased aggregate friction and interlocking, while outdoor exposure caused cracks to widen to roughly twice that of Immersion and W&D specimens.
- The limiting degradation or stabilization level under fatigue cycles, identified by the point when transverse displacement remained stable beyond a certain number of fatigue cycles, Ns, was calculated as 2.1 million cycles for W&Df, 1.7 million cycles for Immf, 1.5 million cycles for Outdoorf, and 1.8 million cycles for Controlf specimens. At this level, specimens experienced reductions in strength and stiffness of ~15–18% due to crack growth, diminished aggregate interlock, and bond weakening, after which the degradation mechanism could not progress further.
- Deflection, rather than ultimate strength, governs the serviceability performance of GFRP-RC beams under combined fatigue and environmental exposure and should therefore be considered the controlling design criterion. For example, Outdoorf specimens exhibited ~12% higher deflection before reaching SLS compared to Controlf.
- Continuous alkaline exposure leads to more pronounced stiffness degradation (~18%) than cyclic wet–dry conditions (~12%); therefore, conservative durability factors are recommended for GFRP-RC elements in permanently alkaline or submerged environments.
- The observed stabilization of damage beyond a threshold number of cycles suggests that fatigue design of GFRP-RC beams may consider a stabilized degradation stage rather than progressive failure, particularly for serviceability-based assessments.
- Crack-width control is essential in outdoor exposure conditions, as crack widening governs serviceability earlier than strength degradation, with crack widths increasing up to 0.9 mm, roughly 2× the width observed under Immersion or W&D conditions.
- The present study shows that fatigue cycles could influence the performance of an FRP-RC beam. Adverse environmental conditions could further degrade the performance. However, such beams eventually attain a stabilization point under the application of fatigue cycles when the damage does not advance anymore. The study demonstrates that FRP-RC beams maintain robust load-carrying capacity under cyclic and monotonic loading, with environmental conditions influencing deformability and ultimate load. Deflection and crack widths under serviceability limit states were quantified, and stabilization points under fatigue cycles were determined for all specimens.

Future research should further clarify the specific research gaps and innovations associated with FRP-reinforced concrete members under fatigue and environmental exposure, with a clearer distinction from studies focused on externally bonded FRP sheets or plates.

## Figures and Tables

**Table 1:** Bending toughness parameter.

Condition	*I*	*P_ult_* (kN)	*Δ_ult_* (mm)
Control1	8.61	181	15.78
Outdoor1	15.92	176	19.41
Outdoor2	14.45	168	19.73
W&D1	18.94	178	19.00
W&D2	12.1	166	16.90
Imm1	9.12	176	17.00
Imm2	18.45	175	21.43
Controlf	13.17	177	20.70
Immf	13.93	209	18.14
W&Df	17.31	151	18.73
Outdoorf	15.42	192	17.00

**Table 2:** Ratios of the experimental to allowable displacements (δ_exp_/δ_code_).

Condition	*δ_exp_*/*δ_code_*
Immf	92%
W&Df	158%
Outdoorf	96%
Controlf	109%

**Table 3:** Coefficient values (A, B) applied in Equation (4) for deflection-related degradation rate.

Condition Specimen	Coefficients	
	A	B
Controlf	−0.235	1.781
W&Df	−0.177	1.369
Outdoorf	−0.363	2.667
Immf	−0.332	2.476

**Table 4:** Estimation of the number of cycles by control of crack-width.

Condition	Cycles(*1000)	Degradation (mm)
Controlf	440	3
W&Df	Passing the limit of 0.7 mm	
Outdoorf	Passing the limit of 0.7 mm	
Immf	400	3
All conditions	Passing the limit of 0.7 mm	

## Data Availability

The original contributions presented in this study are included in the article. Further inquiries can be directed to the corresponding author.
